# Alterations at the Cross-Bridge Level Are Associated with a Paradoxical Gain of Muscle Function *In Vivo* in a Mouse Model of Nemaline Myopathy

**DOI:** 10.1371/journal.pone.0109066

**Published:** 2014-09-30

**Authors:** Charlotte Gineste, Coen Ottenheijm, Yann Le Fur, Sébastien Banzet, Emilie Pecchi, Christophe Vilmen, Patrick J. Cozzone, Nathalie Koulmann, Edna C. Hardeman, David Bendahan, Julien Gondin

**Affiliations:** 1 Aix-Marseille Université, Centre National de la Recherche Scientifique (CNRS), Centre de Résonance Magnétique Biologique et Médicale (CRMBM) Unité Mixte de Recherche (UMR), Marseille, France; 2 Department of Physiology, Institute for Cardiovascular Research, VU University Medical Center, Amsterdam, The Netherlands; 3 Institut de Recherche Biomédicale des Armées, Physiologie des activités physiques, Brétigny sur Orge, France; 4 School of Medical Sciences, University of New South Wales, Sydney, Australia; University of Minnesota, United States of America

## Abstract

Nemaline myopathy is the most common disease entity among non-dystrophic skeletal muscle congenital diseases. The first disease causing mutation (Met9Arg) was identified in the gene encoding α-tropomyosin_slow_ gene (*TPM3*). Considering the conflicting findings of the previous studies on the transgenic (Tg) mice carrying the *TPM3* ^Met9Arg^ mutation, we investigated carefully the effect of the Met9Arg mutation in 8–9 month-old Tg(*TPM3*)^Met9Arg^ mice on muscle function using a multiscale methodological approach including skinned muscle fibers analysis and *in* *vivo* investigations by magnetic resonance imaging and 31-phosphorus magnetic resonance spectroscopy. While *in* *vitro* maximal force production was reduced in Tg(*TPM3*)^Met9Arg^ mice as compared to controls, *in* *vivo* measurements revealed an improved mechanical performance in the transgenic mice as compared to the former. The reduced *in* *vitro* muscle force might be related to alterations occuring at the cross-bridges level with muscle-specific underlying mechanisms. *In vivo* muscle improvement was not associated with any changes in either muscle volume or energy metabolism. Our findings indicate that *TPM3*(Met9Arg) mutation leads to a mild muscle weakness *in* *vitro* related to an alteration at the cross-bridges level and a paradoxical gain of muscle function *in* *vivo*. These results clearly point out that *in* *vitro* alterations are muscle-dependent and do not necessarily translate into similar changes *in* *vivo.*

## Introduction

Nemaline myopathy (NM), the most common form of congenital myopathies, is a genetic muscular disorder characterized by muscle weakness and rod-shaped structures in skeletal muscle [1]. NM has been classified into six different subtypes, ranging from neonatal-lethal forms to late only slowly-progressive, based on clinical manifestations including the severity of muscle weakness and the age at onset [2]. To date, nine genes, most of them encoding proteins associated with muscle thin filaments, have been identified as causing NM in humans [3], [4]. The first disease causing mutation (Met9Arg) has been identified in the gene encoding α-tropomyosin_slow_ (*TPM3*) in a large Australian autosomal dominant family with childhood onset NM [5].

Striated muscle tropomyosin (Tm) is a fundamental component of the thin filament and forms an α-helical coiled-coil dimer which binds to actin and the troponin (Tn) complex. Three major isoforms of Tm have been reported in skeletal muscle: α-Tm_fast_, β-Tm and α-Tm_slow_ [6]. In vertebrates, the level of each isoform varies among muscles and Tm is formed as a homodimer or heterodimer, although the preferred pairing is an α/β heterodimer [7], [8]. Interestingly, the level of β-Tm was reduced in patients carrying the Met9Arg mutation leading to a switch from α/β heterodimers to a predominance of α/α dimers. As a consequence, the mutant protein is incorporated into the thin filament, especially in muscles exhibiting a complete type I fibers predominance, *i.e*. where α-Tm_fast_ is inevitably absent [9]. Accordingly, patient with type 1 fibers predominance had a more severe muscle phenotype in comparison with a patient with a mixed population of type 1 and 2 fibers [9]. Considering that Tm plays a key role in the regulation of skeletal muscle contraction by controlling the Ca^2+^ sensitivity of force and by modulating the kinetics of actin–myosin cross-bridge cycling [10], it has been suggested that the changes in dimeric species related to the Met9Arg mutation might exert a poisoning effect on skeletal muscle function [11]. Although recent studies reported an alteration of cross-bridge cycling kinetics in other *TPM3*-based NM [12], [13], the effect of Met9Arg substitution on human skeletal muscle fiber function has never been investigated probably due to the paucity of muscle biopsy material.

On that basis, a transgenic mouse model expressing the dominant negative Met9Arg human mutation in *TPM3* was generated more than ten years ago in order to improve our knowledge on the mechanisms involved in NM-induced muscle weakness [14]. These mice develop nemaline bodies within skeletal muscle fibers and a late-onset muscle weakness (*i.e*. starting at 5–6 months of age), the latter being determined on the basis of a standard whole-body strength test. Surprisingly, single EDL muscle fibers analysis from Tg(*TPM3*)^Met9Arg^ mice failed to reveal significant difference in specific force production as compared to controls. Moreover, maximal tetanic force of the gastrocnemius (GAS) muscle measured *in* *situ* at optimum muscle length was not different between Tg(*TPM3*)^Met9Arg^ and control mice while a minor force impairment has been reported at shorter (*i.e*. below optimum) muscle length for the transgenic mice [15]. These conflicting findings may arise from the methodology used in the previous investigations that might have precluded firm conclusion about the effect of Met9Arg mutation on mouse muscle function. Indeed, the previous single fiber study has been conducted on a very small number of EDL muscle fibers (*i.e*. ranging from 7 to 12 fibers) [14] and *in* *situ* investigations of Tg(*TPM3*)^Met9Arg^ mouse GAS muscle function have been performed on 2.5–3.5 month-old Tg(*TPM3*)^Met9Arg^ mice [15], *i.e*. an age where no muscle weakness was detected on the basis of whole-body strength test. Furthermore it should be pointed out that the *in* *vivo* and *in* *vitro* studies performed on 8 month-old mice (*i.e*. an age where muscle weakness was detected) were conducted on different muscles (EDL *vs*. forearm muscle respectively) [14]. Alternatively, considering that Tg(*TPM3*)^Met9Arg^ mice have limited type 1 fiber predominance, as compared to patients, it has been suggested that the presence of α-Tm_fast_ and the possible interaction with β-Tm would allow the formation of α/β heterodimers, thereby leading to a preserved skeletal mouse muscle function [11]. Overall, it remained to determine whether and to which extent the Met9Arg mutation affects both *in* *vitro* and *in* *vivo* muscle function.

Considering the different methodological approaches of the previous studies, we aimed at carefully investigating the effect of the Met9Arg mutation on the muscle function of 8–9 month-old Tg(*TPM3*)^Met9Arg^ mice through a straightforward methodological approach of both *in* *vivo* and *in* *vitro* analyses which would allow us to robustly compare the effects of Met9Arg mutation. *In vitro* experiments have been performed on a large sample of skinned fibers of EDL and GAS muscles (*i.e*. ranging from 40 to 50 fibers per muscle and per group). For the first time, both *in* *vivo* and *in* *vitro* experiments were performed on the same muscle (*i.e*. gastrocnemius) of 8 month-old mice (*i.e*. an age where muscle weakness was previously detected) so that we could provide a comprehensive picture of the skeletal muscle phenotype of the Tg(*TPM3*)^Met9Arg^ mouse model. We originally characterized strictly non-invasively the anatomical, functional and metabolic GAS muscle function of Tg(*TPM3*)^Met9Arg^ mice using magnetic resonance imaging (MRI) and 31-phosphorus magnetic resonance spectroscopy (^31^P-MRS).

## Materials and Methods

### Animals

Tg(*TPM3*)^Met9Arg^ mice and control littermates (wild-type, WT) were used for the experiments conducted in agreement with the French guidelines for animal care and in conformity with the European convention for the protection of vertebrate animals and institutional guidelines n° 86/609/CEE November 24, 1986. All animal experiments were approved by the Institutional Animal Care Committee of Aix-Marseille University (permit number: #15-14052012). Experiments were performed on eight- to nine –month-old mice given that, on the basis of whole-body strength test, muscle weakness has been reported to occur at 6 months of age [14]. Mice were housed in an environment-controlled facility (12–12 hour light-dark cycle, 22°C), received water and standard food *ad libitum*. They were identified through PCR genotyping from mouse tail DNA as previously described [14].

### *In vivo* experiments: GAS muscle

#### Animal preparation

Mice were initially anesthetized in an induction chamber using 4% isoflurane in 33% O_2_ (0.5 L/min) and 66% N_2_O (1 L/min). The left hindlimb was shaved before an electrode cream was applied at the knee and heel regions to optimize electrical stimulation. Each anaesthetized mouse was placed supine in a home-built cradle which has been specially designed for the strictly noninvasive functional investigation of the left hindlimb muscles [16]. Throughout a typical experiment, anesthesia was maintained by gas inhalation through a facemask continuously supplied with 1.75% isoflurane in 33% O_2_ (0.2 L/min) and 66% N_2_O (0.4 L/min). Exhaled and excess gases were removed through a canister filled with activated charcoal (Smiths Industries Medical System, Sheffield, UK) mounted on an electrical pump extractor (Equipement Vétérinaire Minerve, Esternay, France). Physiological temperature was adjusted with an electrical heating blanket. The foot was positioned on the pedal of the ergometer with a variable ankle joint angle. The hindlimb was centered inside a 20 mm-diameter ^1^H Helmholtz imaging coil and the belly of the GAS muscle was located above an elliptical (8×12 mm) ^31^P-MRS surface coil. Muscle contractions were achieved by transcutaneous electrical stimulation using two rod-shaped 1.5 mm-diameter surface electrodes integrated in the cradle and connected to an electrical stimulator (type 215/T; Hugo Sachs Elektronik-Harvard Apparatus GmbH, March-Hugstetten, Germany). One electrode was placed at the heel level and the other one was located just above the knee joint. The GAS muscle was chosen because it is easily accessible for ^31^P-MRS measurements and preferentially activated by our *in* *vivo* experimental set-up [16].

#### Study design

Mice were tested twice over a one-week period in order to assess mechanical performance, muscle volume and metabolic changes during a standardized stimulation protocol of the whole GAS muscle *in vivo*.

During the first testing session, GAS transcutaneous stimulation was first elicited with square-wave pulses (0.5 ms duration). The individual maximal stimulation intensity was determined on the basis of a progressive stimulation intensity increase until there was no further peak twitch force increase. This intensity was then maintained to elicit tetanic stimulation at 150 Hz (0.75 sec duration; n = 14 for WT group; n = 24 for Tg(*TPM3*)^Met9Arg^ group). The corresponding measurements were performed at three different ankle joint angles (90°, neutral position, the footplate of the ergometer perpendicular to the tibia; 70°, *i.e*. at long muscle length and 120°, *i.e*. at short muscle length) in order to take into account potential length-dependent muscle weakness [15]. A resting period of 10 minutes was considered between each measurement.

During the second testing session, MRI measurements were performed at rest to get information about muscle volume. Additionally, metabolic changes were investigated using ^31^P-MRS during a standardized stimulation protocol (n = 13 for WT group; n = 19 for Tg(*TPM3*)^Met9Arg^ group) consisting of 6 min of repeated single twitch isometric contractions delivered at a frequency of 1.7 Hz [16] with an ankle joint angle of 90°.

#### Force output measurements

Isometric force of the whole GAS muscle *in vivo* was measured with a home-built ergometer consisting of a 9×24 mm foot pedal coupled to a force transducer. The transducer was constructed by sticking a strain gauge (ref 1-LY11-6/120A; HBM GmbH, Darmstadt, Germany; 120-ohm internal resistance) on a Bakelite slat (0.4 mm thickness) in a Wheatstone bridge design (3×120 ohm). Electrically-evoked muscle contractions led to a deformation of the bakelite slat transmitted by the foot pedal resulting in a change in the strain gauge electrical resistance and a proportional voltage change. The resulting output signal was amplified with a home-built amplifier (Operational amplifier AD620; Analog Devices, Norwood, MA, USA; gain = 70 dB; bandwidth = 0–5 kHz) and converted to a digital signal (PCI-6220; National Instruments, Austin, TX, USA). It was continuously monitored and recorded on a personal computer using the WinATS software (Sysma, Aix-en-Provence, France).

#### MR acquisition

Investigations were performed in a 4.7-Tesla horizontal superconducting magnet (47/30 Biospec Avance, Bruker, Karlsruhe, Germany) equipped with a Bruker 120-mm BGA12SL (200 mT/m) gradient insert.

For MR imaging, ten contiguous axial slices (thickness = 1 mm), covering the region from the knee to the ankle, were acquired at rest using a spin echo sequence (TE = 10.6 ms; TR = 1000 ms; one accumulation; field of view = 42×30 mm; matrix size = 256×192; acquisition time = 3 min 12 sec).

For ^31^P-MRS measurements, spectra (8-kHz sweep width; 2048 data points) from the GAS region were continuously acquired at rest and throughout the standardized stimulation protocol. A fully relaxed spectrum (12 accumulations, TR = 20 sec) was acquired at rest followed by a total of 256 free induction decays (FID) (TR = 1.875 sec). The first 64 FIDs were acquired at rest and summed together. The next 192 FIDs were acquired during the stimulation period and were summed by packets of 32, allowing a temporal resolution of ∼60 sec.

### *In vitro* experiments: skinned fibers

#### Preparation of skinned fibers

EDL and GAS muscles were dissected and stored in a relaxing solution (in mM; 100 BES, 6.97 EGTA, 6.48 MgCl_2_, 5.89 Na-ATP, 1 DTT, 40.76 K-propionate, 14.5 creatine phosphate (CP), pH 7.1 at 15°C) containing 50% glycerol at −20°C. Isolated single muscle fibers were dissected from the isolated muscles in a 50% glycerol/relaxing solution and skinned for 15 min in a 50% glycerol/relaxing solution containing 1% Triton X-100 at ∼4°C. Each single skinned fiber was mounted using aluminum T clips between a length motor (ASI 403A, Aurora Scientific Inc, Ontario, Canada) and a force transducer element (ASI 315C-I, Aurora Scientific Inc) in a skinned fiber apparatus (ASI 802D, Aurora Scientific Inc) that was mounted on an inverted microscope (Zeiss Axio Observer A1).

Sarcomere length (SL) was set and dimensions of the muscle fibers (width and diameter) were measured, using a high speed VSL camera with calibrated ASI 900B software (Aurora Scientific Inc). Width and diameter of the fibers were measured at three points along the fiber using a X40 objective of an inverted microscope and a custom made prism that was mounted in the bath and the cross-sectional area was determined assuming an elliptical cross-section.

Three different types of bathing solutions were used during the experimental protocols: a relaxing (in mM: 100 BES, 6.97 EGTA, 6.48 MgCl_2_, 5.89 Na-ATP, 1 dithiothreitol (DTT), 40.76 K-propionate, 14.5 CP, 0.24 mM PMSF, 0.01 mM E64); a pre-activating solution with low EGTA concentration (in mM: 100 BES, 0.1 EGTA, 6.42 MgCl_2_, 5.87 Na-ATP, 1 DTT, 41.14 K-propionate, 14.5 CP, HDTA 6.9, 0.24 mM PMSF, 0.01 mM E64); and an activating solution (in mM: 100 BES, 7.0 CaEGTA, 6.28 MgCl_2_, 5.97 Na-ATP, 1 DTT, 40.64 K-propionate, 14.5 CP, 0.24 mM PMSF, 0.01 mM E64). The temperature of the bathing solutions was kept constant during the whole protocol at 20°C using a temperature controller (ASI 825A, Aurora Scientific Inc).

### Mechanical measurements of skinned fibers

Considering that the previous single fiber study has been conducted on a very small number of EDL fibers (*i.e*. ranging from 7 to 12 fibers) and that previous *in vivo* and *in vitro* studies have been performed on different muscles (EDL *vs*. forearm muscle respectively) [14], we performed a whole set of *in vitro* measurements on a large sample of both WT and Tg(*TPM3*)^Met9Arg^ skinned EDL and GAS muscle fibers (*i.e*. ranging from 40 to 50 fibers per group), that included force-pCa relation, rate of force redevelopment measurements and muscle fiber stiffness (see below).

To assess force-SL relation, the maximal force generated at various SL (ranging from 2.0 to 3.5 µm) was determined. Mechanical experiments were performed at a SL of ∼2.5 µm for EDL and GAS skinned muscle fibers from both groups (WT and Tg(*TPM3*)^Met9Arg^).

To determine force-pCa relation, skinned muscle fibers were sequentially bathed in solutions with pCa values (pCa = −log of molar free Ca^2+^ concentration) ranging from 4.5 to 9.0 and the steady-state force was measured. Maximal force was determined by dividing the force generated at pCa 4.5 by CSA.

Rate of force redevelopment (*k*_tr_) was measured by using the large slack/release approach [17] to disengage force-generating cross-bridges from the thin filaments, which were isometrically activated. Fast activation of the fiber was achieved by transferring the skinned muscle fibers from the pre-activation solution containing a low concentration of EGTA (pCa 9.0) to a pCa 4.5 activating solution. Once the steady-state was reached, a slack equivalent to 30% of the muscle length was rapidly induced at one end of the muscle using the motor. This was followed immediately by an unloaded shortening lasting 30 msec. The remaining bound cross-bridges were mechanically detached by rapidly restretching the muscle fiber to its original length, after which force redevelops.

#### Skinned muscle fiber stiffness

Single fibers were maximally activated at pCa 4.5. The muscle length was changed in a step-like fashion (either increased or decreased) by ±0.3, ±0.6, ±0.9, and ±1.2% of muscle length when the fiber reached a steady-state isometric force (*F*_0_). Stiffness, associated with the number of strongly bound cross-bridges prior to stretch, was estimated from the relationship between length change (Δ*L*) and the peak force response (*F*_1_).

### Biochemical and Molecular analyses

#### Intracellular ATP concentration

Mice were anesthetized intra-peritoneally with a pentobarbital injection (50 mg/kg). GAS muscles were harvested and freeze-clamped with liquid nitrogen-chilled metal tongs before mice were submitted to a cervical dislocation. The corresponding tissue sample was used in order to measure intracellular ATP concentration. Water soluble metabolites were extracted from 40–50 mg of freeze-clamped GAS muscle sample using perchloric acid solution (0.6 M) as previously described [18]. ATP concentration was determined using a bioluminescence assay according to the manufacturer’s instructions (ATP Determination Kit (A22066), Invitrogen, Eugene, Oregon, USA).

#### MHC composition of single skinned fibers

After mechanical recordings, each fiber was placed in tube containing a buffer solution and stored at −80°C. For the EDL muscle fibers, MHC composition was determined as previously described [19]. For GAS muscle fibers, single fibers were subjected to MHC isoform analysis using a SDS-PAGE electrophoretic method. Each fiber was placed in 20 µl of a myosin extraction solution containing (mM): NaCl 300, NaH_2_PO_4_ 100, Na_2_HPO_4_ 50, Na_4_P_2_O7 10, MgCl_2_.6H_2_O 1, EDTA 10 and 2-β-mercaptoethanol 1.4; pH 6.5. After 24 h incubation at 4°C, the extract was diluted with 20 µl glycerol and stored at –20°C until required for the separation process. Electrophoresis was performed using a Mini Protean II system (Biorad, Marnes-la-Coquette, France). The separating gel solution contained 30% glycerol, 8% acrylamide–bis (50∶1), 0.2 M Tris, 0.1 M glycine and 0.4% SDS. The stacking gel was composed of 30% glycerol, 4% acrylamide-bis (50∶1), 70 mM Tris, 4 mM EDTA and 0.4% SDS. Then 10 µl extract were denatured using 10 µl buffer containing 5% 2-β-mercaptoethanol, 100 mM Tris base, 5% glycerol, 4% SDS and bromophenol blue, for 3 min at 100°C. Samples were loaded onto vertical gels, whilst two lanes were loaded with MHC extract from a mix of control muscles known to contain the four adult MHC isoforms. Gels were run at constant voltage (72 V) for 31 h and then silver-stained. The MHC protein isoform bands were scanned using a densitometer (GS-700, Biorad, Marnes-la-Coquette, France). The MHC isoforms in single-fibres were identified by comparing them with bands of myosin extracts from control muscle.

### Troponin-I Western blot expression

GAS muscle samples from WT (n = 4) and Tg(*TPM3*)^Met9Arg^ (n = 4) mice were solubilized as described previously [20]. In brief, frozen muscle samples were homogenized in a liquid nitrogen cooled mortar and re-suspended in 1 ml cold 10% trichloracetic acid (TCA) solution dissolved in acetone containing dithiotheitol (DTT) (0.2% w/v) and stored at −80 degrees for 1 h. Subsequently, homogenates were brought to room temperature stepwise: 20 min at −20°C, 20 min at 4°C and 20 min at room temperature (while being mixed on a vortex in between all steps). Then, muscle homogenates were centrifuged at 12.000 g for 15 min followed by washing the tissue pellets with 1 mL of 0.2% w/v DTT-acetone solution and shaking them for 5 min at room temperature. This cycle of centrifugation, washing and shaking was repeated five times. Tissue pellets were freeze-dried overnight and homogenized in 1-D sample buffer containing 15% glycerol, 62.5 mM Tris (pH 6.8), 1% w/v SDS and 2% w/v DTT (final concentration 5 ug dry weight/uL).

To discriminate between both Tn-I_slow_ and Tn-I_fast_ isoforms, 4 uL of muscle homogenates in 1-D sample buffer (20 ug of dry weight tissue) were loaded on a 15% acrylamide SDS-PAGE gel. Subsequently, the gel ran first for 20 min at 200 V and thereafter 160 min at 400 volt at 15°C. After completion of the run, the gel was blotted for 90 min at constant amperage of 320 mA using a semidry blotting system (Trans-Blot SD Cell, Bio-Rad, USA). After staying overnight in blocking solution (5% milk in TBS-T), the blot was incubated at room temperature for 1.5 h in a pan-specific antibody directed against Tn-I (#4002, Cell Signaling, USA) (1∶500 in blocking solution), washed for 30 min and put in secondary antibody goat anti-rabbit HRP (Dako, Denmark) (1∶2500 in blocking solution) for 1 h at room temperature. Thereafter, the blot was washed with TBS-T for 30 min and treated with ECL prima reagens (GE Healthcare, UK) for 5 minutes. The blot was scanned using a LAS 3000 (Fujifilm Medical Systems, USA).

### Data processing

#### Mechanical performance

For *in vivo* measurements (whole muscle) maximal isometric peak force was calculated for each ankle joint angle. Maximum rate of force development (in mN/ms) and the half relaxation time, *i.e*. the time to obtain half of the decline in maximal tetanic force, were also calculated. Regarding the standardized stimulation protocol, the force time integral (FTI; mN.sec/mm^2^) of each contraction was calculated and then summed together.

For all stimulation protocols, force was divided by the corresponding maximal hindlimb muscles CSA (see below) in order to obtain specific force (in mN/mm^2^).

For *in vitro* measurements (skinned fibers), the steady state force was measured for each calcium concentration. Measured force values were normalized to the maximal force obtained at pCa 4.5. The obtained force-pCa data were fitted to the Hill equation providing pCa_50_ (calcium concentration giving 50% of the maximal force) and Hill coefficient, *n*_H_ (index of myofilament cooperativity). A single exponential fit was used to determine the rise of force concerning *k*_tr_ measurements. Finally, stiffness was determined from the slope of the linear regression between *F*_1_ and Δ*L.*

#### MR data

The maximal CSA was determined from the largest slice and the hindlimb muscles volume (in mm^3^) was calculated as the sum of the five cross-sectional areas of the six consecutive largest slices using a proprietary software developed using IDL (Interactive Data Language, Research System, Inc., Boulder, CO, USA). ^31^P-MRS data were processed using a proprietary software developed using IDL [21]. Relative concentrations of phosphocreatine (PCr), inorganic phosphate (Pi) and ATP were obtained with a 60 sec time-resolution by a time-domain fitting routine using the AMARES-MRUI Fortran code and appropriate prior knowledge of the ATP multiplets. Absolute amounts of phosphorylated compounds were expressed relative to a resting ATP concentration determined *in* *vitro* (see above). PCr to ATP ratios were calculated from the peak areas of the fully relaxed spectrum. Intracellular pH (pHi) was calculated from the chemical shift of the Pi signal relative to PCr [22].

### Statistical analyses

Statistical analyses were performed with Statistica software version 9 (StatSoft, Tulsa, OK, USA). Normality was checked using a Kolmogorov-Smirnov test. Two-factor (group × time) analysis of variance (ANOVAs) with repeated measures on time were used to compare isometric force production, metabolites concentrations and pHi. Two-factor (group × contraction number or stimulation frequency) ANOVAs with repeated measures on contraction number or stimulation frequency were used to compare force production. When a main effect or a significant interaction was found, Newman–Keuls *post-hoc* analysis was used. Student’s unpaired *t* tests were used for other comparisons. Data are presented as mean ± standard error of mean (SEM). Significance was accepted when *P*<0.05.

## Results

### *In vivo* experiments

#### Anatomical measurements

Body weight was not different (*P*>0.05) between WT (31.7±1.4 g) and Tg(*TPM3*)^Met9Arg^ (33.8±1.7 g) groups. Both maximal CSA and muscle volume were similar (*P*>0.05) in the two groups (34.7±1.2 mm^2^ *vs*. 32.6±0.8 mm^2^; 156±5 mm^3^ *vs.* 147±4 mm^3^ for WT and Tg(*TPM3*)^Met9Arg^ groups, respectively).

#### Mechanical performance

**Figure 1:**
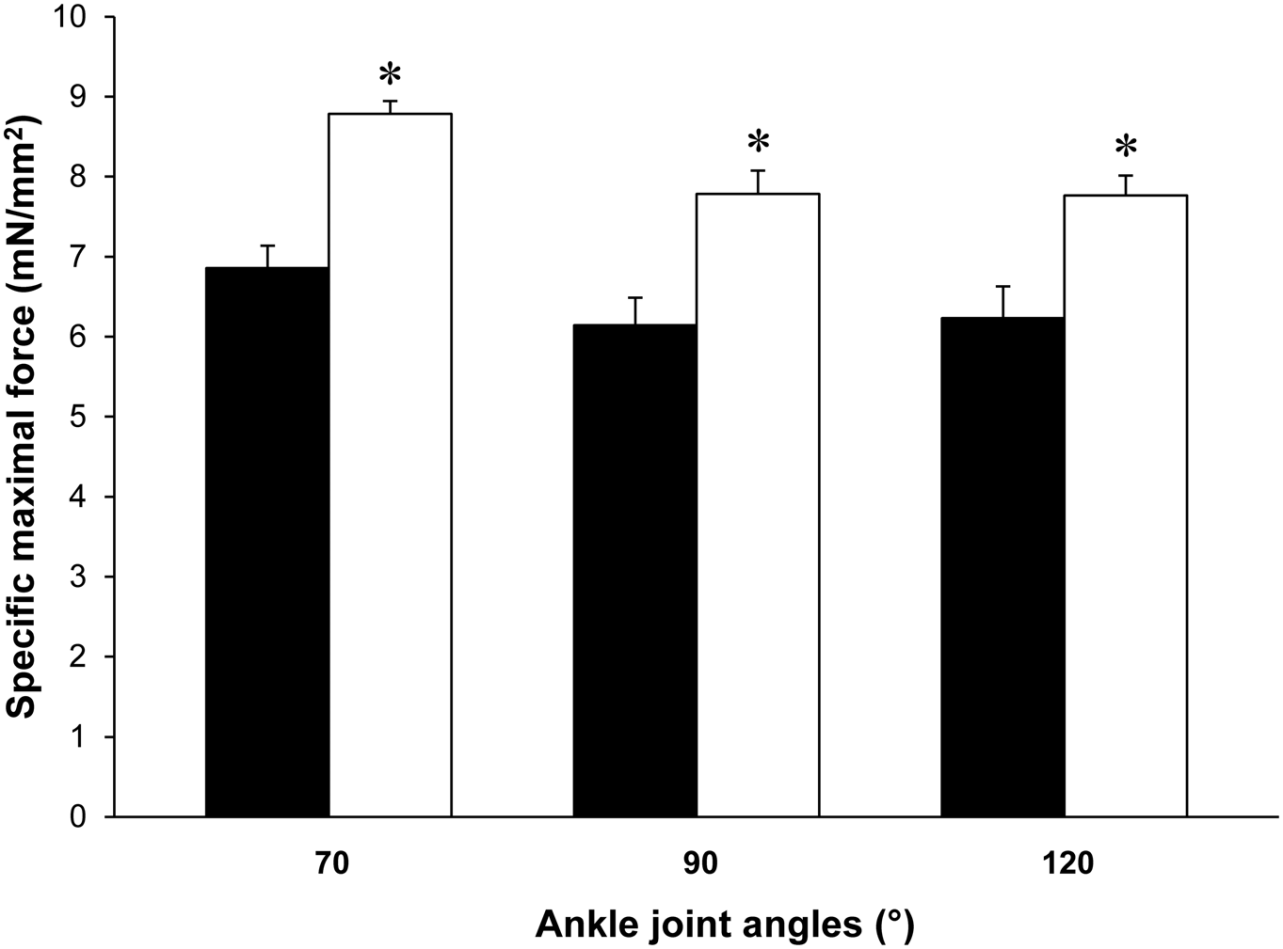
*In vivo* specific maximal force production measured at short (70°), neutral (90°) and long (120°) muscle length. Note that force was always higher in Tg(*TPM3*)^Met9Arg^ mice (○, n = 24) as compared to WT mice (•, n = 14) whatever the muscle length (or ankle joint angle). Values are presented as the mean ± SEM. Significantly different between groups *P<0.05.

A 20% increase (*P*<0.05) in absolute maximal tetanic force was quantified in Tg(*TPM3*)^Met9Arg^ mice as compared to WT mice. When normalized to muscle CSA, maximal specific force was ∼30% higher (*P*<0.05) in the Tg(*TPM3*)^Met9Arg^ than in the WT group at neutral (7.78±0.16 mN/mm^2^ *vs.* 6.14±0.28 mN/mm^2^, respectively), short (8.79±0.29 mN/mm^2^ *vs.* 6.86±0.35 mN/mm^2^, respectively) and long (7.76±0.25 mN/mm^2^ *vs.* 6.23±0.40 mN/mm^2^, respectively) muscle length (Figure 1). When normalized to muscle volume, maximal force was also 30% higher (*P*<0.05) in Tg(*TPM3*)^Met9Arg^ group relative to WT group at neutral (1.72±0.04 mN/mm^3^ vs. 1.32±0.05 mN/mm^3^, respectively), short (1.97±0.06 mN/mm^3^ vs. 1.47±0.07 mN/mm^3^, respectively) and long (1.79±0.05 mN/mm^3^ vs. 1.37±0.09 mN/mm^3^, respectively) muscle length, indicating that force normalization using either muscle volume or maximal CSA leads to similar differences between the two groups. Considering the absence of muscle-length dependence on the corresponding differences between the two groups, only mechanical measurements performed at a 90° ankle joint angle were considered for the subsequent analyses.

The maximum rate of force development was 20% faster (*P*<0.001) in the Tg(*TPM3*)^Met9Arg^ mice (2.68±0.09 mN/ms) as compared to WT mice (2.24±0.07 mN/ms), while the half relaxation time was similar (*P*>0.05) in Tg(*TPM3*)^Met9Arg^ mice (198±5 ms) and WT mice (201±6 ms).

**Figure 2:**
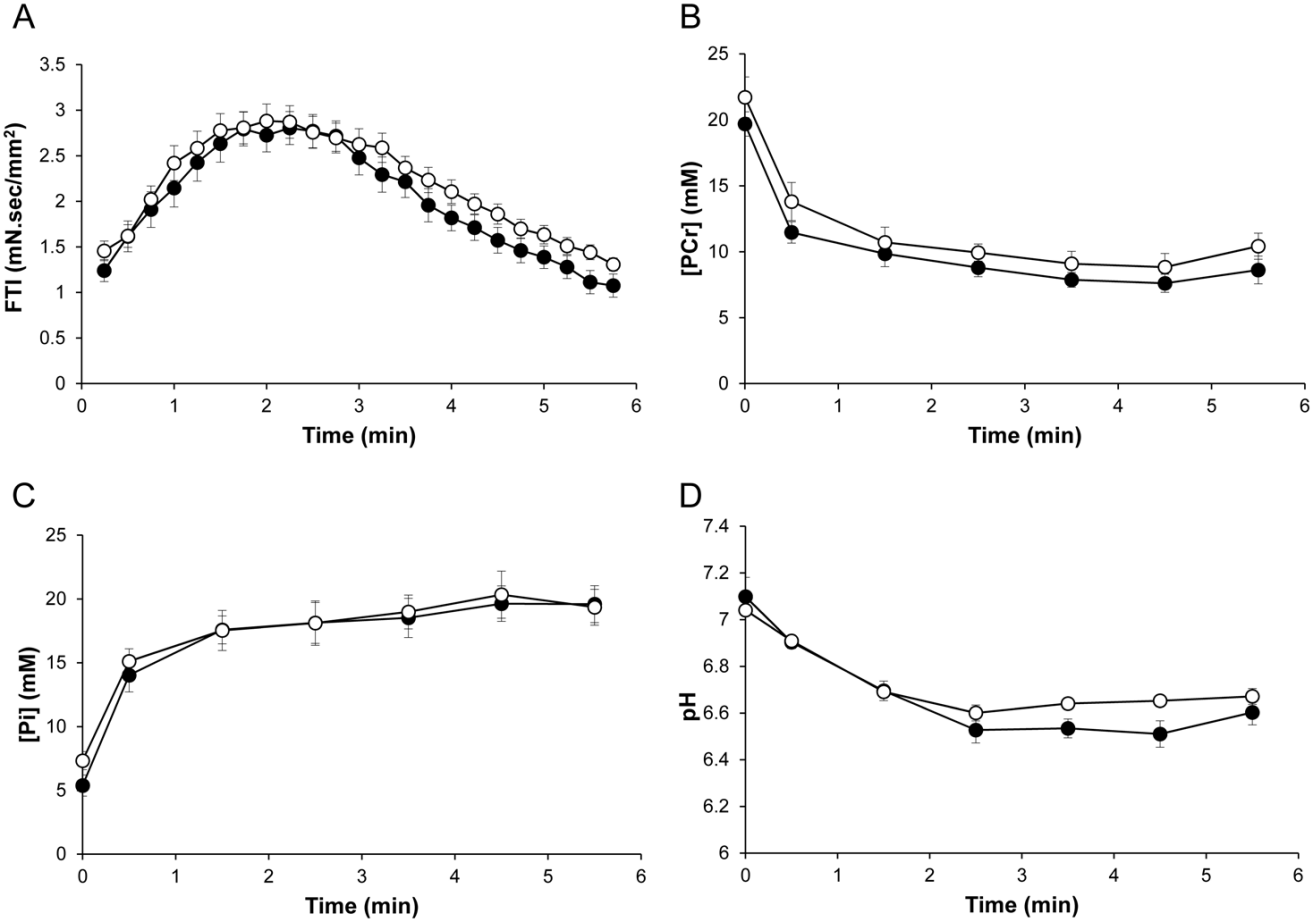
Force time integral (FTI, A) and changes in gastrocnemius [PCr] (B), [Pi] (C), pHi (D) during the stimulation protocol. Note that neither FTI nor metabolic variations were different between Tg(*TPM3*)^Met9Arg^ mice (○, n = 19) and WT mice (•, n = 13). Values are presented as mean ± SEM.

FTI during the standardized 6-min stimulation protocol was similar (*P*>0.05) for Tg(*TPM3*)^Met9Arg^ mice (2.18±0.13 mN.sec/mm^2^) and WT mice (1.99±0.15 mN.sec/mm^2^) (Figure 2A).

#### Metabolic changes

[PCr]/[ATP] resting ratios were similar (*P*>0.05) in Tg(*TPM3*)^Met9Arg^ (2.36±0.17) and WT groups (2.12±0.10). For both groups, [PCr] fell rapidly throughout the fatigue protocol and reached a similar steady state at the end of the stimulation (*P*>0.05) (Figure 2B). As expected, the [Pi] time-course evolved as a mirror of the [PCr] time-dependent changes for both groups, and increased during the fatigue protocol until it reached a plateau after 3 min of exercise (Figure 2C). At rest, pHi was not significantly different (P>0.05) in WT (7.10±0.08) and Tg(*TPM3*)^Met9Arg^ mice (7.04±0.02). pHi decreased throughout the stimulation session so that the acidosis extent was identical for the two groups at the end of the fatigue protocol (Figure 2D). Taken together, the fatigue protocol-induced metabolic changes and FTI were comparable in the two groups, thereby suggesting a similar energy cost of contraction.

### *In vitro* experiments

**Figure 3:**
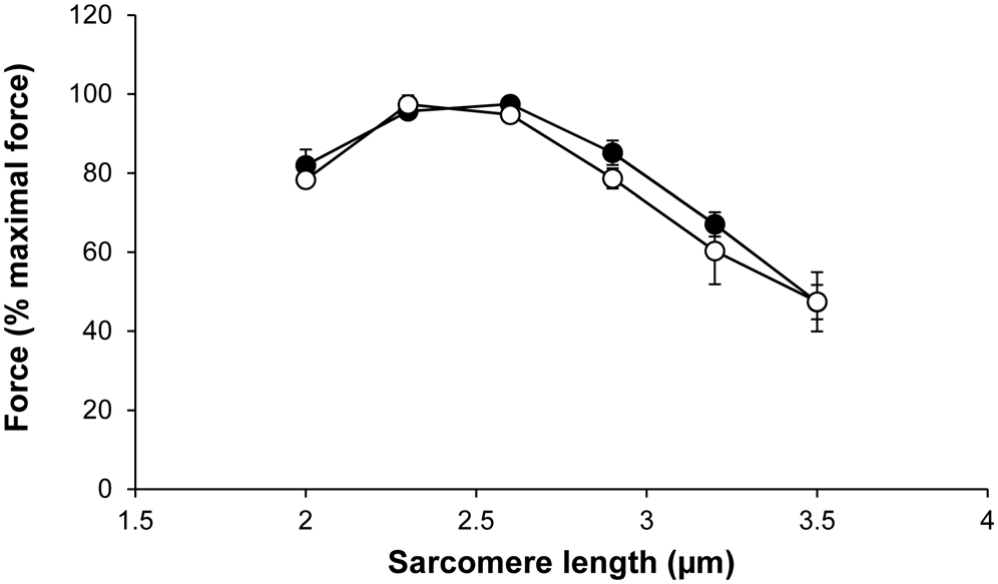
Force-sarcomere length relation. Mean relative force-sarcomere length relationships obtained from GAS Tg(*TPM3*)^Met9Arg^ (○) and WT (•) muscle fibers. Both curves overlap and display a characteristic force plateau followed by a comparable descending limb. Values are presented as the mean ± SEM.

WT and Tg(*TPM3*)^Met9Arg^ force-SL relations showed that both curves overlapped for EDL (data not shown) and GAS (Figure 3) muscle fibers. The optimal thick-thin filament overlap for EDL and GAS muscle fibers was at 2.5 µm for WT and Tg(*TPM3*)^Met9Arg^ groups, so that experiments were performed at this SL for both groups.

#### Maximal force

**Figure 4:**
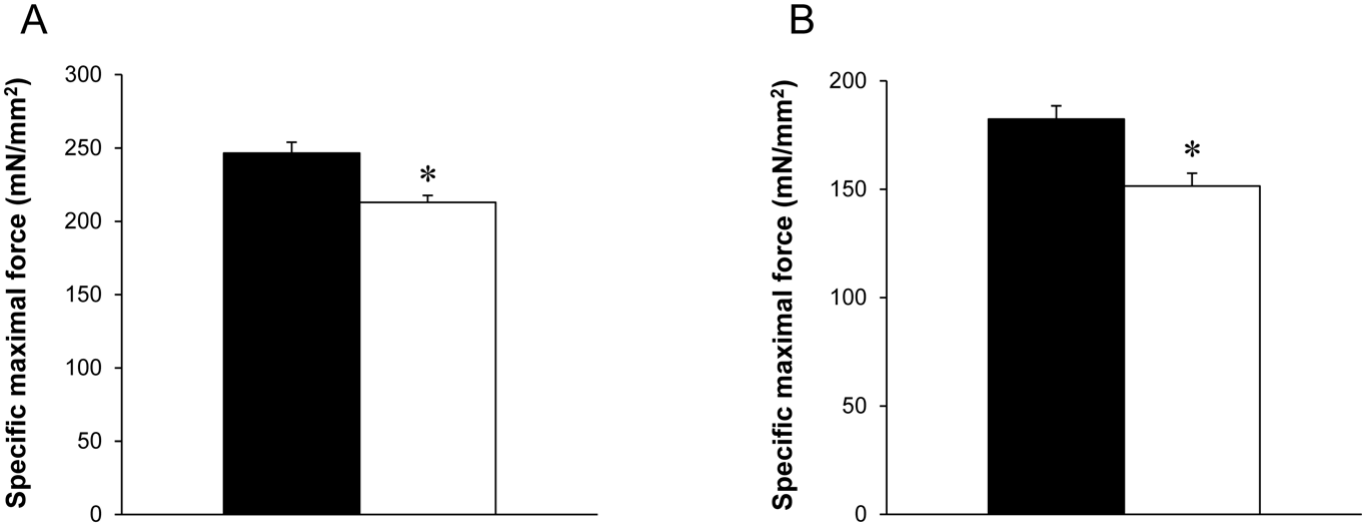
*In vitro* specific maximal force production obtained at pCa 4.5 from GAS (A) and EDL (B) skinned muscle fibers at optimal sarcomere length. Maximal isometric steady-state force generation was significantly lower in Tg(*TPM3*)^Met9Arg^ (○) as compared to WT fibers (•) for both GAS (n = 49 for WT and n = 50 for Tg(*TPM3*)^Met9Arg^) and EDL muscles (n = for 50 WT and n = 48 for Tg(*TPM3*)^Met9Arg^). Values are presented as the mean ± SEM. Significantly different between groups *P<0.05.

Specific maximal force (*i.e*. recorded at pCa 4.5) was 14% lower (*P*<0.05) in GAS Tg(*TPM3*)^Met9Arg^ fibers (213±5 mN/mm^2^; n = 50) relative to GAS WT fibers (247±7 mN/mm^2^; n = 49) (Figure 4A). Interestingly, specific maximal force was also 17% lower in EDL (Tg(*TPM3*)^Met9Arg^ fibers (152±6 mN/mm^2^_;_ n = 50) relative to EDL WT fibers (183±6 mN/mm^2^; n = 48) (Figure 4B).

#### Force-pCa relation

**Figure 5:**
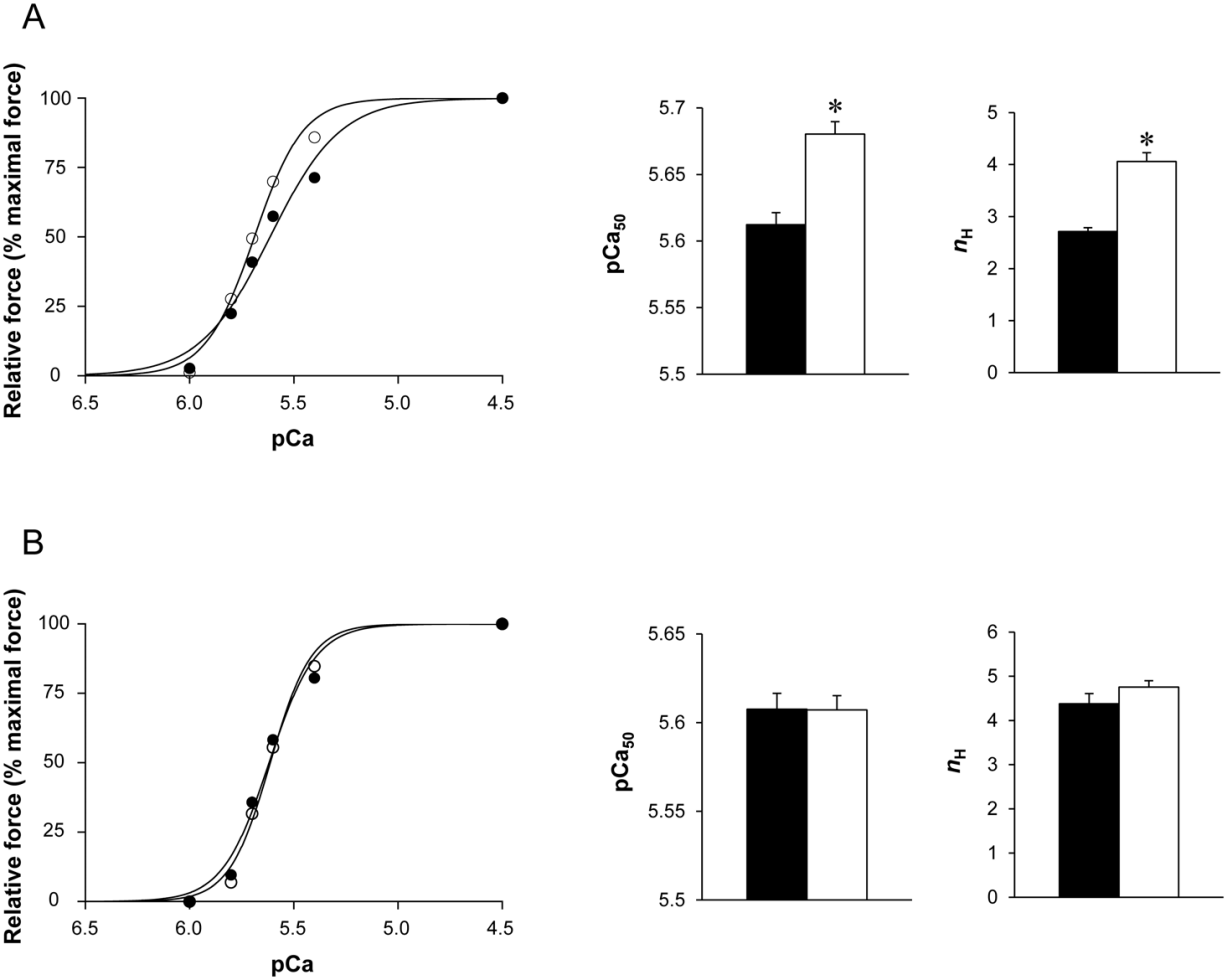
Force-Ca^2+^ relationship obtained from GAS (A) and EDL (B) skinned muscle fibers. Force was generated in response to incubation with incremental increase of [Ca^2+^]. Note the slight leftward shift of the force–Ca^2+^ relationship in GAS Tg(*TPM3*)^Met9Arg^ fibers (○, n = 50) as compared to GAS WT fibers (•, n = 49) (left panel Fig. A), resulting in a higher pCa_50_ values and *n*_H_ value (right panel Fig. A) in Tg(*TPM3*)^Met9Arg^ fibers (ΔpCa_50_ = 0.07; middle panel Fig. A). On the contrary, both curves were overlapped in EDL muscle fibers (n = for 50 WT and n = 48 for Tg(*TPM3*)^Met9Arg^, left panel Fig. B), resulting in a similar pCa_50_ value (middle panel Fig. B) and *n*_H_ value (right panel Fig. B). Values are presented as the mean ± SEM. Significantly different between groups *P<0.05.

The force–pCa relation was slightly shifted to the left in GAS Tg(*TPM3*)^Met9Arg^ fibers, resulting in a significantly higher (*P*<0.05) pCa_50_ values in GAS Tg(*TPM3*)^Met9Arg^ fibers (5.680±0.009) when compared to the GAS WT fibers (5.612±0.009; *i.e*. ΔpCa_50_ = 0.07) (Figure 5A). On the contrary, no difference was observed in the force–pCa curve of EDL muscle. Accordingly, pCa_50_ was similar for the two groups (*i.e*. 5.607±0.008 *vs.* 5.607±0.009 for the Tg(*TPM3*)^Met9Arg^ and WT groups, respectively; Figure 5B).

*n* _H_: Tg(*TPM3*)^Met9Arg^ fibers had a significantly higher *n*_H_ (4.06±0.17) as compared to WT fibers (2.71±0.07) for GAS muscle, whereas this parameter was not different between the two groups for EDL (4.38±0.23 for WT fibers *vs*. 4.75±0.15 for Tg(*TPM3*)^Met9Arg^ fibers).

#### *k*_tr_ measurements

**Figure 6:**
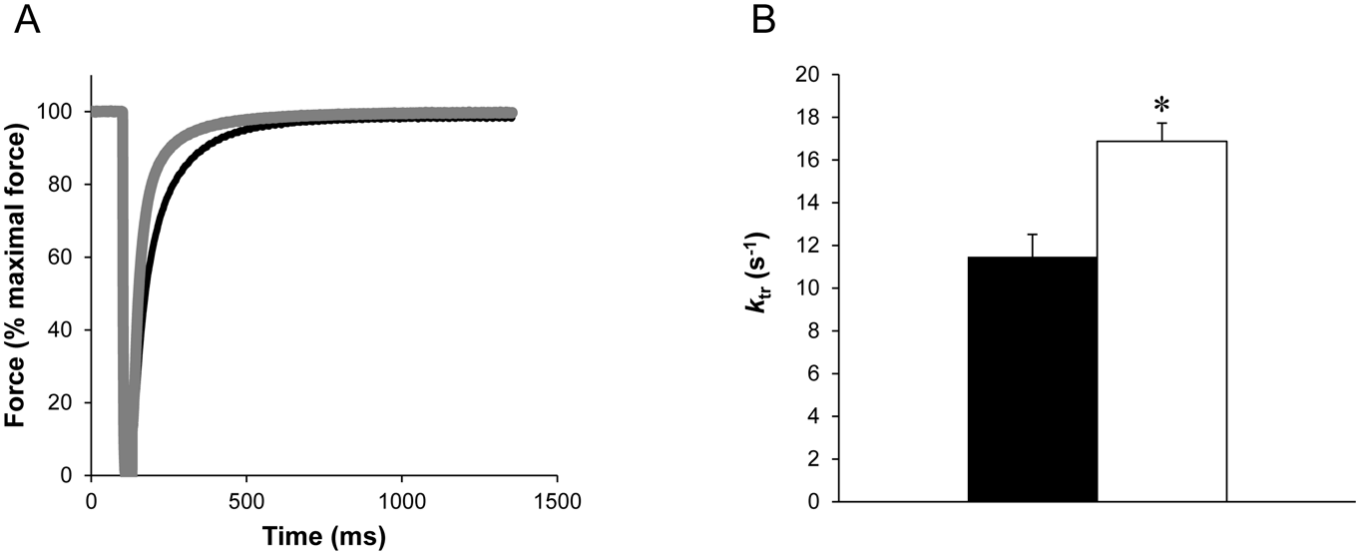
*k*_tr_ measurements. Example of *k*_tr_ measurement at pCa 4.5 with superimposed the results of a GAS Tg(*TPM3*)^Met9Arg^ fiber (grey) and a GAS control fiber (black) (A). Note that *k*_tr_ value was increased in Tg(*TPM3*)^Met9Arg^ fibers (○, n = 26) as compared to WT fibers (•, n = 27) (B). Values are presented as the mean ± SEM. Significantly different between groups *P<0.05.

*k*_tr_ was significantly higher (*P*<0.05) for GAS Tg(*TPM3*)^Met9Arg^ fibers (16.9±0.9 s^−1^; n = 54) when compared to the GAS WT fibers (11.5±1.0 s^−1^; n = 54) (Figure 6). *k*_tr_ was not significantly different (*P*>0.05) between WT (22.2±1.4 s^−1^; n = 36) and Tg(*TPM3*)^Met9Arg^ (22.7±0.9 s^−1^; n = 31) fibers for the EDL muscle.

#### Muscle fiber stiffness

Active stiffness was similar (*P*>0.05) in GAS WT (72.2±3.3 mN/mm^2^/% fiber length change) and GAS Tg(*TPM3*)^Met9Arg^ (74.3±2.3 mN/mm^2^/% fiber length change) fibers. No difference (*P*>0.05) was also observed in EDL WT (66.2±3.1 mN/mm^2^/% fiber length change) and EDL Tg(*TPM3*)^Met9Arg^ (58.8±2.8 mN/mm^2^/% fiber length change) fibers. As a result, the force/stiffness ratio was significantly lower (*P*<0.05) in GAS Tg(*TPM3*)^Met9Arg^ fibers (2.8±0.1) as compared to GAS WT fibers (3.2±0.1), and in EDL Tg(*TPM3*)^Met9Arg^ fibers (2.4±0.1) as compared to EDL WT fibers (2.9±0.1).

### Molecular analyses

**Figure 7:**
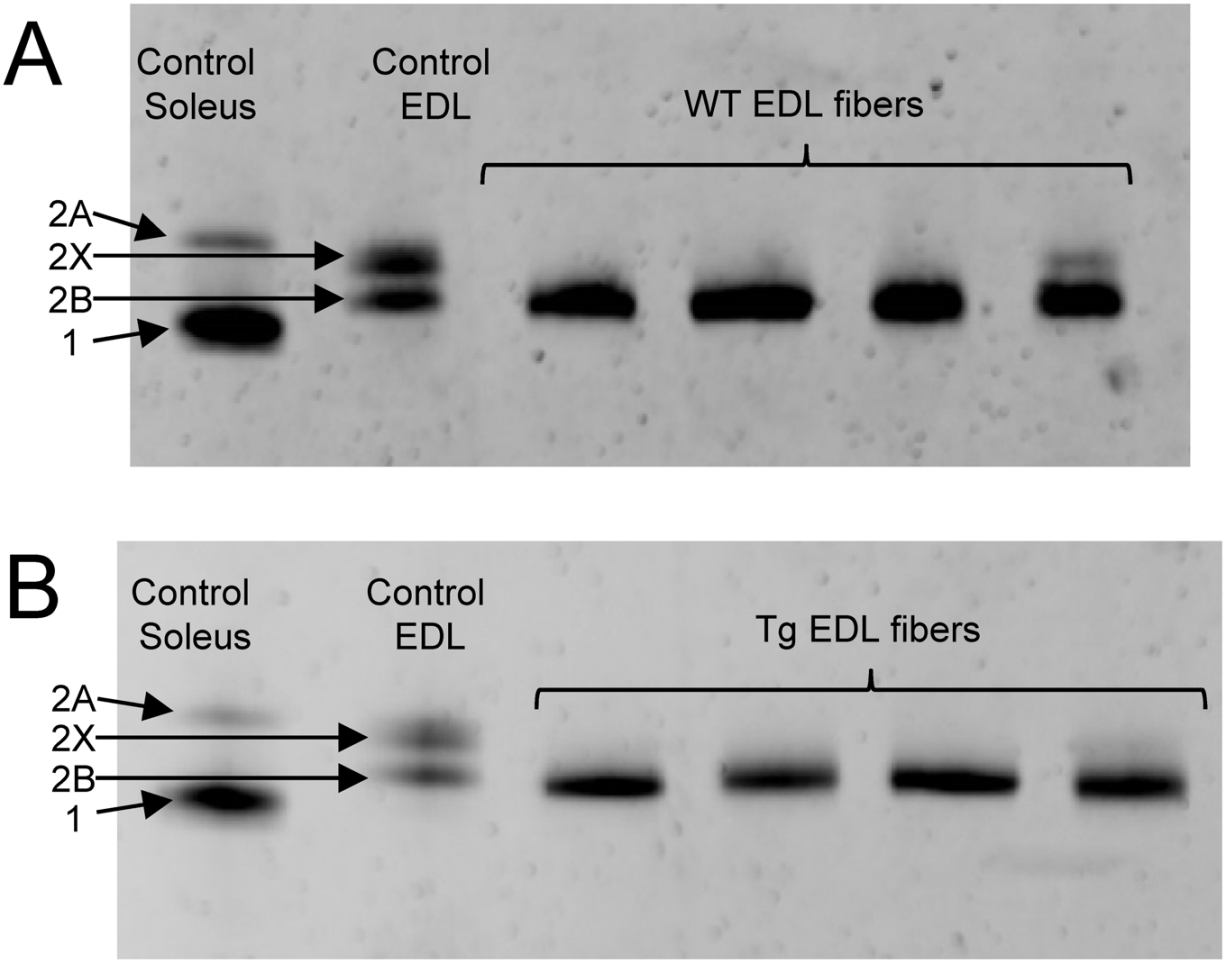
Example of gel electrophoresis results. MHC composition of some EDL WT (A) and Tg(*TPM3*)^Met9Arg^ (B) tested fibers. Note that all the fibers expressed only MHC 2B isoforms.

**Figure 8:**
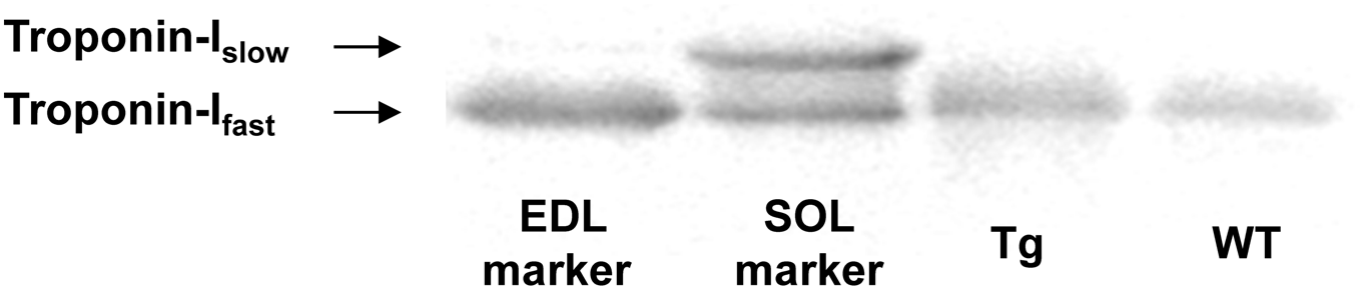
Example of Troponin-I isoform expression results. Western blot example including a muscle homogenate from the EDL that expresses predominanlty Tn-I_fast_ isoform, a SOL homogenate that expresses both Tn-I_slow_ and Tn-I_fast_, and GAS muscle from a Tg(*TPM3*)^Met9Arg^ and a WT mouse. Western blot analysis revealed that GAS muscle from Tg(*TPM3*)^Met9Arg^ (*n = *4) and WT mice (*n = *4) expressed exclusively Tn-I_fast_ isoforms.

ATP concentration in GAS muscles was similar in WT (9.3±0.5 mM) and Tg(*TPM3*)^Met9Arg^ (9.2±0.5 mM) groups. The gel electrophoresis revealed that all the analyzed GAS and EDL WT and Tg(*TPM3*)^Met9Arg^ fibers expressed only MHC 2B isoforms (Figure 7). In addition, Tn-I expression level showed that GAS muscles from both Tg(*TPM3*)^Met9Arg^ and WT mice expressed only Tn-I_fast_ isoform (Figure 8).

## Discussion

In this study, we aimed at providing a comprehensive picture of the skeletal muscle phenotype of Tg(*TPM3*)^Met9Arg^ mice through a combination of both *in* *vitro* and *in* *vivo* analyses. On the basis of a multidisciplinary and multiscale methodological approach, we originally reported that muscle function related to the Met9Arg mutation was abnormal. Surprisingly, the corresponding results recorded *in* *vivo* and *in* *vitro* were strikingly opposite. Indeed, whereas *in* *vitro* maximal force production was reduced in Tg(*TPM3*)^Met9Arg^ mice, *in* *vivo* measurements illustrated an improved mechanical performance. These *in* *vivo* findings were not associated with any change in either muscle volume or energy metabolism. Interestingly, the *in* *vitro* alterations involved in the reduced maximal force production were muscle-dependent.

### Met9Arg mutation improved *in* *vivo* muscle function

An unexpected finding of the present study was that *in* *vivo* specific maximal force was 30% higher in Tg(*TPM3*)^Met9Arg^ mice. This result is clearly opposite to the initial findings reported for this mouse model, *i.e*. a muscle weakness starting at 6 months of age and progressing until 12 months of age [14]. This discrepancy could actually be related to the methodological approach. Corbett *et al.* used a standard whole-body strength test, similar to the human Gower’s maneuver, which mainly involved forearm muscles and was also dependent of the mice motivation [14]. On the contrary, we specifically measured GAS muscle force production using an original experimental set-up recently developed in our laboratory [16]. Another potential explanation for this discrepancy could be related to a different amount of Met9Arg protein in GAS and forearm muscles. However, Corbett *et al.* reported a similar amount of mutant protein in both muscle groups [14]. De Haan *et al*. observed that maximal tetanic force of the GAS muscle measured *in* *situ* at optimum muscle length was not different between Tg(*TPM3*)^Met9Arg^ and control mice but slightly reduced at shorter (*i.e*. below optimum) muscle length [15]. Our results clearly indicated that *in* *vivo* GAS muscle force production was increased in Tg(*TPM3*)^Met9Arg^ as compared to controls independently of the muscle length. It is noteworthy that we performed our measurements in 8–9 months mice, an age at which, according to Corbett *et al* [14], muscle weakness is present whereas De Haan *et al*. investigated muscle function in younger mice (*i.e*. 2–3 months of age) [15]. This age difference might explain, at least in part, the corresponding discrepancies. Taken together, our *in* *vivo* results did not support the previous findings of a negative effect of Met9Arg mutation on *in* *vivo* GAS muscle but rather showed an increased contractile function in these transgenic mice.

This improved *in* *vivo* muscle function in Tg(*TPM3*)^Met9Arg^ mice cannot be related to muscle hypertrophy given that our MRI investigations indicated that hindlimb muscles volume was not different between the two groups. Our results are consistent with those from Corbett *et al*. indicating that the hypertrophy of fast glycolytic fibers occurred in Tg(*TPM3*)^Met9Arg^ mice between two and 6 months of age but was partially reversed between 6 and 12 months of age [14], even though no statistical analysis was performed on the corresponding data.

Finally, ^31^P-MRS investigations showed that PCr consumption, Pi production and acidosis were not different between transgenic and control mice throughout the stimulation protocol. Considering that FTI during exercise was similar between the two groups, the energy cost of contraction was unaltered in the Tg(*TPM3*)^Met9Arg^ group. While this result is in contradiction with the higher energy cost of contraction observed in two mouse models of severe form of NM carrying *ACTA1* mutations [23], [24], our findings are consistent with the lack of changes in the expression of genes encoding the proteins involved in metabolic pathways and further support the hypothesis that defects in energy consumption in NM might only be related to a severe phenotype [25]. Overall, our combined MRI and ^31^P-MRS investigations showed that the improved muscle function in the Tg(*TPM3*)^Met9Arg^ groups cannot be related to either changes in muscle volume or in energy metabolism.

### Met9Arg mutation altered *in* *vitro* muscle function

Our single skinned muscle fibers analysis showed that maximal active force was ∼15% lower in Tg(*TPM3*)^Met9Arg^ group as compared to WT group and so for both EDL and GAS muscles. To our knowledge, only one study investigated single EDL muscle fiber function in these transgenic mice and the authors showed no significant difference in maximal force between similarly-aged (nine-month old) Tg(*TPM3*)^Met9Arg^ and control fibers [14]. However, it is noteworthy that the previous study only investigated a very small number of muscle fibers (*i.e*. ranging from 7 to 12) [14] while we performed the single fibers analysis in a large sample size (*i.e*. ranging from 40 to 50 fibers) on both EDL and GAS muscles. On that basis, one could reasonably assume that the previous methodological approach has inevitably precluded firm conclusion on the effect of Met9Arg mutation on *in* *vitro* mouse muscle function. Interestingly, Ottenheijm *et al*. reported a severe muscle fiber weakness in patients with *TPM3*-based NM (60 to 90% reduction in force production) [13] whereas Ochala *et al*. reported a preserved maximal force but an altered force production at submaximal activation [12]. Taken together, these findings indicated that each *TPM3* mutation specifically affects muscle function and that the Met9Arg mutation induces a mild muscle fiber weakness *in* *vitro*.

It is well acknowledged that *in* *vitro* specific force is mainly determined by the number of attached cross-bridges and the force produced by each cross-bridge. Considering that muscle stiffness was not different between control and transgenic mice, the reduced specific force and the related decreased force-to-stiffness ratio in both EDL and GAS Tg(*TPM3*)^Met9Arg^ muscle fibers indicated that the lower force-generating capacity is predominantly caused by a decrease in the force per cross-bridges rather than a reduction in the number of cross-bridges. Interestingly, other muscle-dependent mechanisms could be involved in Met9Arg mutation-induced muscle weakness.

At saturating calcium concentration, *k*_tr_ reflects the myosin cross-bridge cycling turnover rate and, according to the two-state cross-bridge model, is proportional to the attachment rate *f*_app_ + *g*_app_, with *f*_app_ being the rate constant for attachment and *g*_app_ the rate constant for detachment. Interestingly, it is known that an increased attachment/detachment rates, illustrated by an increased *k*_tr_, results in weakly-bound cross-bridges having a weak affinity for actin [26]. Of interest, the increased number of weakly-bound cross-bridges resulting from cardiac thin filament mutations has been related to the disruption of the stabilization of the steric blocking-state (B-state) and the formation of the closed-state (C-state) [27], [28]. These alterations have also been associated to an increase calcium sensitivity [29], [30]. It should be pointed out that Tm overlap regions are essential for the assembly of the N-term tail of troponin T [31], which is required to maintain the thin filament in the steric B-state equilibrium [32], [33]. The *TPM3*(Met9Arg) mutation is located at the tropomyosin end–end overlap area and is supposed to bind to F86 in troponin T [34], *i.e*. a residue of the T1 fragment of Tn-T which is located close to the N-term end of Tn-T and bound close to the C-term end of Tm [35]. Moreover, a reduced maximal force generating capacity and an elevated calcium sensitivity have been reported in a recent study on human hypertrophic cardiomyopathy caused by cardiac sarcomeric mutations and related to the destabilization of the B-state formation [36]. Therefore, one might suggest that the *TPM3*(Met9Arg) mutation likely impairs formation of the B-state in the GAS muscle by increasing the number of weakly bound cross-bridges and attachment/detachment rates, leading to both a reduced force production and an elevated Ca^2+^ sensitivity. The increased Ca^2+^ sensitivity was not related to any shift in Tn-I isoform expression [37] given that both Tg(*TPM3*)^Met9Arg^ and WT mice expressed only the Tn-I_fast_ isoform. The increased cooperativity we observed in transgenic GAS muscle fibers also indicates that the switching of the Tm is increased. Given that an increased attachment/detachment rates results in weakly-bound cross-bridges [26], we suggested that the switching of Tm occurs only from the B-state to C-state. Our result are in accordance with a recent study showing that the conformational changes of actomyosin were disturbed and that the formation of the strong-binding conformational state of the myosin head was inhibited in a patient with congenital myopathy carrying the E117K mutation in β-tropomyosin [38].

Surprisingly and contrarily to Tg(*TPM3*)^Met9Arg^ GAS muscle fibers, Ca^2+^-sensitivity, cooperativity and *k*_tr_ were unaltered in EDL muscle fibers of transgenic mice as compared with controls, thereby indicating that the effects of Met9Arg mutation are muscle-dependent. Our results are in accordance with the unaltered calcium sensitivity previously reported in EDL muscle fibers from the same Tg(*TPM3*)^Met9Arg^ mice. In addition, a similar cooperativity was observed in psoas muscle from a NM mouse model carrying nebulin mutation [39], while this parameter was decreased in the tibialis cranialis muscle [40]. It should be pointed out that our *in* *vitro* experiments have been performed on both EDL and GAS muscle fibers expressing only MHC 2B isoforms and a similar amount mutant Tm [14], so that any methodological bias related to either muscle fiber type composition or amount of mutant Tm isoform expression could be ruled out. Although, our data clearly show that the effects of the *TPM3*(Met9Arg) mutation are muscle-specific, the underlying mechanisms remain to be determined. These results clearly highlighted that the effects of TPM3-based NM can be heterogeneous among muscles and should thereby be considered for the design of therapeutic interventions.

It has been demonstrated that Met9Arg-α-Tm_slow_ is incorporated into the skeletal muscle thin filament and can form dimers *in* *vitro* with itself, with WT-α-Tm_slow_ and with β-Tm [9], [11]. In patients with α-Tm_slow_-based NM, α-Tm_slow_ is the predominant isoform so that the Tm filament must be composed predominantly of α-Tm_slow_ homodimers containing the mutant Tm. Accordingly, patient with type 1 fibers predominance had a more severe muscle phenotype in comparison with a patient with a mixed population of type 1 and 2 fibers [9]. Although, it has been demonstrated that both EDL and GAS muscles are expressing the mutant Tm which contributes to ≥93% of the total α-Tm_slow_ protein in these two muscles [25], one might suggest that muscle phenotype might be more severe in a slow-twitch muscle such as the soleus as compared to the fast-twitch muscles GAS and EDL. However, a similar combination of both *in* *vitro* and *in* *vivo* analyses (*e.g*. ^31^P-MRS and force production measurements) would certainly not be feasible on the soleus muscle. Further investigations are still needed to characterize the effects of the *TPM3*(Met9Arg) mutation on a slow-twitch muscle. We hypothesized that GAS and EDL muscle function of Tg(*TPM3*)^Met9Arg^ mice would be preserved in Tg(*TPM3*)^Met9Arg^ mice given that the predominance of type 1 fibers is low as compared to patients indicating that a significant proportion of α-Tm_fast_/β-Tm heterodimers is maintained (EDL muscle contains 62% of α-Tm_fast_ and 18% of β-Tm [11]). Overall, our results indicated that the Met9Arg-α-Tm_slow_ had a poisoning effect despite the presence of a significant proportion of α-Tm_fast_ and β-Tm isoforms.

### Opposite findings between *in* *vivo* and *in* *vitro* investigations

In the present study, we reported striking divergent findings between *in* *vivo* and *in* *vitro* conditions. The Met9Arg mutation leads to an impaired *in* *vitro* force production whereas we consistently observed a 30% higher *in* *vivo* force generating capacity in Tg(*TPM3*)^Met9Arg^ mice. Considering that our *in* *vivo* and *in* *vitro* investigations were both performed on the GAS muscle of eight- to nine-month old mice, neither muscle type nor age of the mice can explain these divergent findings. Interestingly, although muscle function of nebulin-deficient mice was similarly altered at different levels (*i.e*. skinned muscle fibers, isolated muscles or muscle-tendon-bone unit) [39], [41], [42], other studies reported that *in* *vitro* alterations did not necessarily translate into similar changes *in* *vivo*, and vice versa [23], [43], which should be taken into account for future NM studies.

The mechanisms responsible for this differential effect of Met9Arg mutation on muscle function remain unclear. Although a slight fast-to-slow shift in MHC isoform content has been previously reported in Tg(*TPM3*)^Met9Arg^ [14], it seems unlikely that changes in MHC composition might explain the discrepancy in *in* *vivo* and *in* *vitro* forces. It is well known that maximal isometric force output is typically lower in slow-twitch as compared to fast-twitch fibers [44], [45], [46]. Contrariwise, we observed an increased force production *in* *vivo* for the transgenic mice as compared to controls.

Interestingly, we found that Tg(*TPM3*)^Met9Arg^ mice had a faster maximum rate of force development *in* *vivo* as compared to controls. While this finding may be consistent with the changes in cross-bridges kinetics we observed *in* *vitro*, one could speculate that this faster maximum rate of force development could be also related to a higher passive stiffness of various non-contractile structures, including for instance cytoskeletal proteins, extracellular matrix or tendon, in order to overcompensate for a defective force production at the cross-bridge level. It should be pointed out that increased muscle stiffness and hypertonia has been recently reported in a NM patient with an *ACTA1* mutation [47].

Although we used similar muscles for *in* *vitro* and *in* *vivo* investigations, other variables such as the temperature might explain the discrepancies. Indeed, we measured force at physiological temperature *in* *vivo*, *i.e*. 36°C, whereas isolated fibers experiments were performed at 20°C. However it seems unlikely that this parameter alone could lead to such a difference. On the other hand, one might suggest that the skinning procedure could reduce *in* *vitro* the phosphorylation status of proteins involved in the calcium control, such as phospholamban (PLB). Indeed, unphosphorylated PLB inhibits the SERCA pump [48], [49] while the ability of PLB to inhibit SERCA is lost when PLB is phosphorylated. Therefore, an increase in the phosphorylation status might directly impact SR Ca^2+^ uptake function and muscle contractility. One might suggest that PLB might be more phosphorylated *in* *vivo* as compared to *in* *vitro*, which may lead to faster reuptake of calcium into the SR and therefore increase maximal force, or inversely that PLB might be less phosphorylated *in* *vitro* as compared to *in* *vivo* leading to a slower reuptake and reduced force production *in* *vitro*. The underlying mechanisms remain unclear and further experiments are warranted in to clarify these issues.

In conclusion, we have demonstrated that the *TPM3*(Met9Arg) mutation results in alteration of the skeletal muscle function. We observed conflicting results between *in* *vivo* and *in* *vitro* force measurements for the GAS muscle. While the *in* *vitro* force production was decreased, we reported an increased contractile muscle function *in* *vivo* that might be related to compensatory mechanisms that remain to be determined. Although *in* *vitro* force production was reduced similarly for both EDL and GAS muscle fibers, the underlying mechanisms appear muscle-specific. Overall, the opposite findings between *in* *vivo* and *in* *vitro* investigations and the muscle-specific mechanisms involved in the muscle weakness have to be considered for the design of therapeutic strategies.
